# Enhancement of Colonic Absorptive Function after the Massive Resection of the Small Intestine Based on the Creation of an Artificial Colonic Valve

**DOI:** 10.1038/s41598-020-57865-3

**Published:** 2020-01-21

**Authors:** Gaoyan Deng, Zhijian Deng

**Affiliations:** Department of pediatric surgery, Guangzhou women and children’s medical center, Guangzhou, China

**Keywords:** Intestinal diseases, Intestinal diseases, Nutrition disorders, Nutrition disorders

## Abstract

The colon can have an absorptive function similar to that of the small intestine after the massive resection of the small bowel. To improve colonic absorptive function, we created a valve in the colon (artificial colonic valve, ACV). ACVs were created in 20 rats that had 80 percent of their small intestine resected, with an observation time of 30 weeks. The ACV rats were compared with those in the non-operated control group, the short bowel syndrome (SBS) group and the colon interposition (CI) group. The ACV rats were much heavier than those in the control group, SBS group and CI group. In terms of histology and the levels of α-amylase and the Na^+^-dependent bile salt transporter, the absorptive function of the colons before the valves resembled that of the small intestine. The colonic absorptive function was more obvious in ACV rats than in CI rats. An ACV can enhance colonic absorptive function after the massive resection of the small intestine. The colonic absorptive function of ACV rats was better than that of the rats in the CI group.

## Introduction

**Figure 1:**
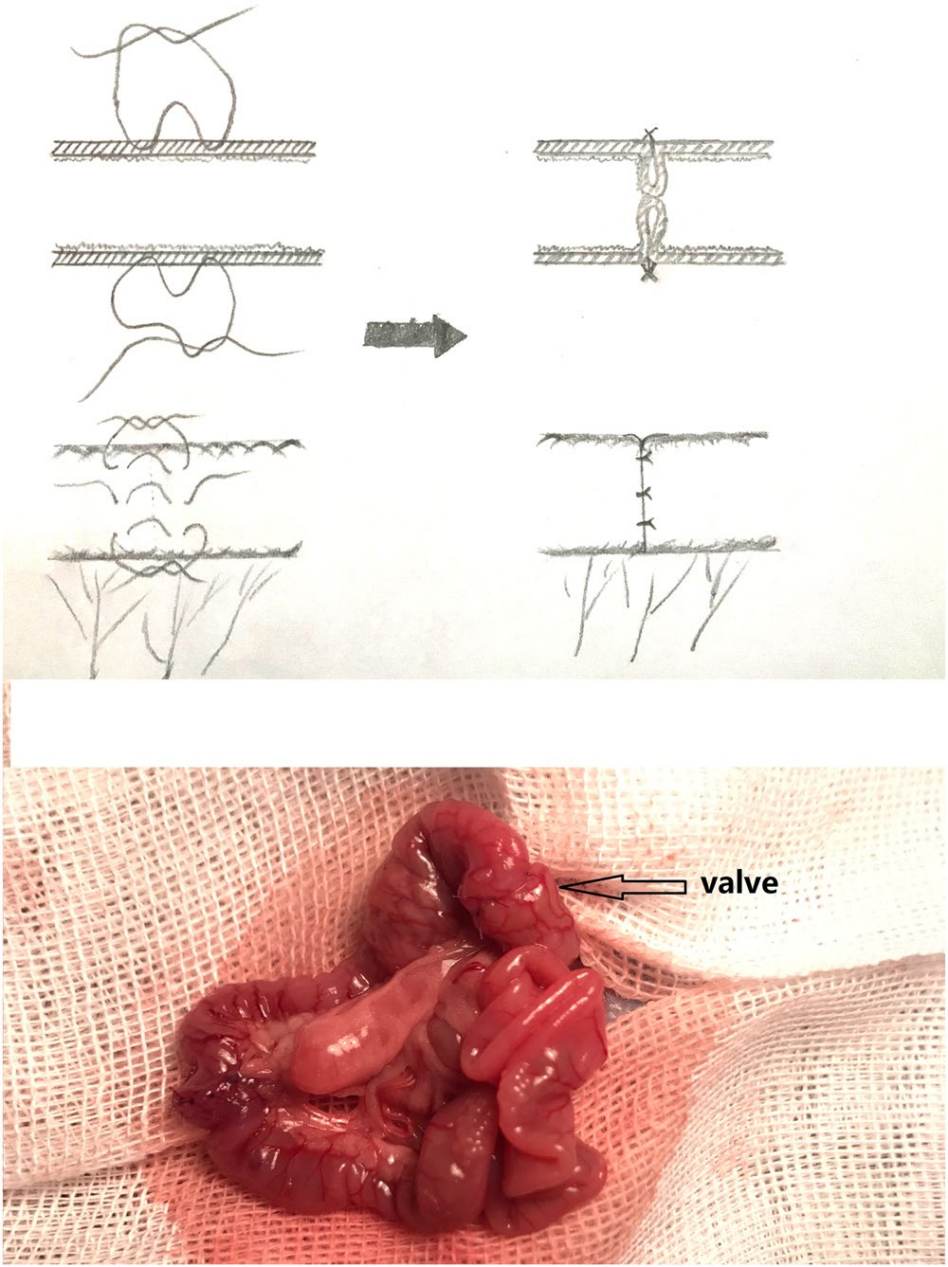
The procedure of making artificial colonic valve(ACV).

Patients with SBS should be treated by a broad expert pool of nutritionists, gastroenterologists and surgeons. Longitudinal intestinal lengthening and tailoring (LILT),^1^ serial transverse enteroplasty (STEP),^2^ segmental reversal of the small bowel^3^ and iso-peristaltic colonic interposition^4^ are the most common surgeries but do not result in an increase in the absorptive area of the intestines. Sequential lengthening procedures and controlled tissue expansion (CTE) may increase the absorptive area.^5^ Distraction enterogenesis is another novel method to increase the absorptive area.^6^ Intestinal transplantation is the last option for patients who have failed intestinal rehabilitation.^7^. Colonic adaptation enables the colon to have an absorptive function similar to that of the small intestine after the massive resection of the small bowel. To improve colonic adaptation, a valve was created in the colon (Fig. 1). Moreover, an ACV can slow the intestinal transit time to enhance the absorption in the small intestine. ACV rats were compared with those in the non-operated control group, SBS group and CI group, with a research time of 30 weeks. The crypt depth, mucosal thickness, percentage of goblet cells, and α-amylase and Na^+^-dependent bile salt transporter levels of the bowels of sacrificed rats were examined.

## Methods

### Experimental design

Eighty Sprague Dawley (SD) rats provided by Guangdong Medical Laboratory Animal Center for this study were divided randomly into 4 groups. The weights of the rats in all the groups were not significantly different (P > 0.5). The rats in the control group (Group 1) received no operation. The SBS rats in Group 2 had 80 percent of their small intestine/ileocaecal valves and caecums resected. The rats in Group 3 had 80 percent of the small intestine/ileocaecal valves and caecums resected, and an ACV was created in the middle colon (the colon before the valve was 5 cm in length). The rats in Group 4 had 80 percent of their small intestine/ileocaecal valves and caecums resected, and CI was performed at the same time (the interposed colon has a length of 5 cm). No food or water was given to the rats from the day before the surgery to the day after the surgery. The rats were anaesthetized by the inhalation of halothane. After 30 weeks, the rats were sacrificed with an intra-cardiac injection of 2 ml pentobarbital. The rats were cared for in accordance with the institutional guidelines of Guangdong Medical Laboratory Animal Center. The study was granted permission by the ethics committee of Guangzhou Women and Children Medical Center.

### Operations

**Figure 2:**
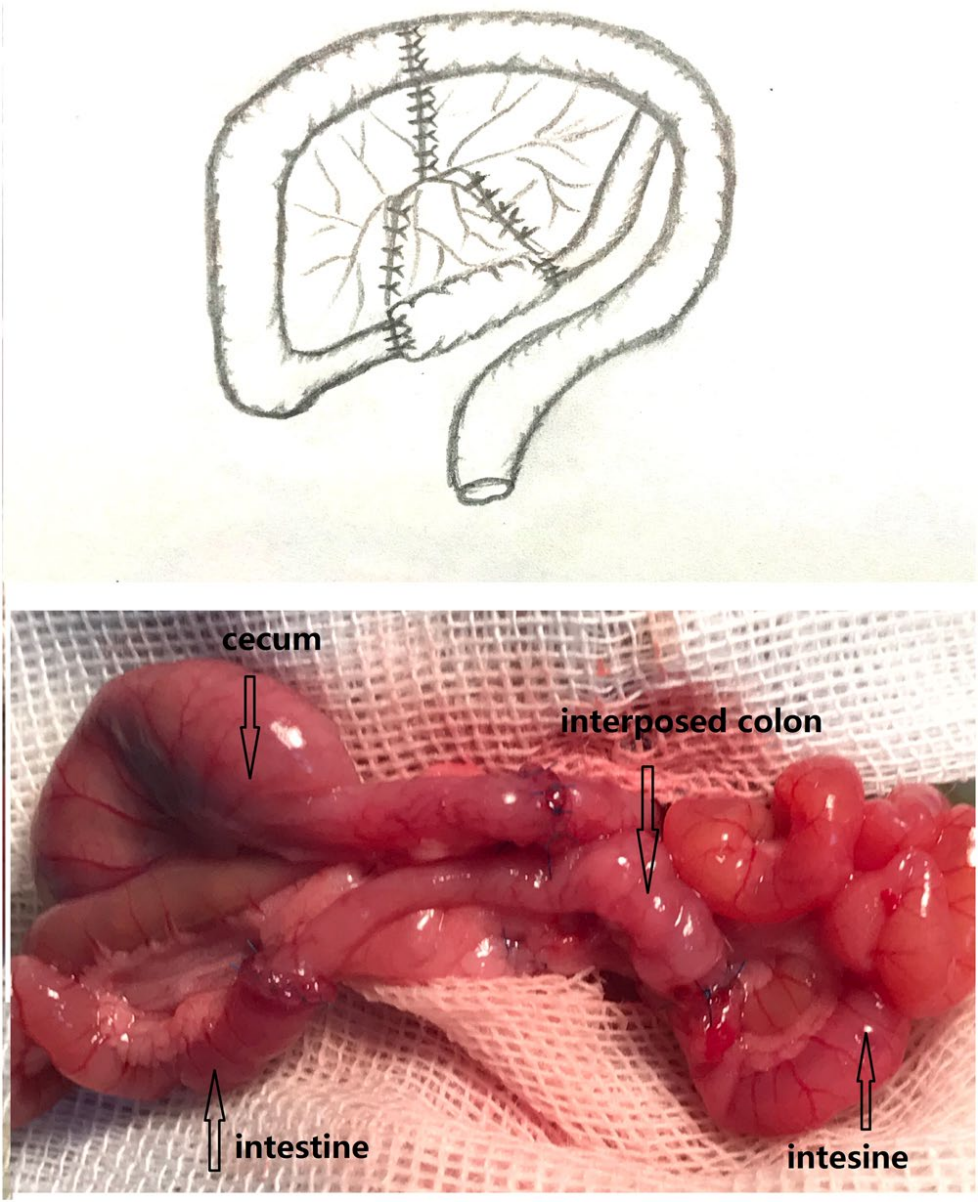
The procedure of colon interposition (CI).

To build the SBS rat model, the rats underwent the resection of the ileum/ileocaecal valves and caecums, leaving 10 cm of the proximal jejunum. In this case, 80 percent of the small intestine was resected. The one-layer end-to-end anastomosis of the remaining jejunum and colon was performed with 6/0 polypropylene sutures. In the ACV procedure, the residual small intestine was anastomosed with the proximal colon after the resection of 80 percent of the small intestine. Eight interruptive sutures were placed onto the sero-muscular colonic layer, each being placed twice separately with a 3 mm distance between the two stitches. After the sutures were tightened up, the colon wall between the two stitches was protruded towards the colonic lumen. Such sutures were uninterruptedly repeated 8 times around the colon. After the sutures were tightened, a valve similar to the ileocaecal valve was formed (Fig. 1). The ACV was created in the middle colon. The colon before the valve was 5 cm in length. During the CI, the interposed colon, with a length of 5 cm (Fig. 2), was taken from the middle of the colon.

### Histological examination

After the rats were sacrificed, specimens were taken from the ileums and colons of the rats in Groups 1 and 2 for full-thickness HE staining and histological examination. For the ACV rats (Group 3), the specimens were taken from sections of the ileum and colon after the valve and from three parts of the colon before the valve (the colon near the original ileocaecal valve is the proximal part of the colon before the valve, the colon near the valve is the distal part of the colon before the valve, and the colon between the two is the middle part of the colon before the valve). For rats in Group 4, the specimens were taken from the ileum, interposed colon and distal colon. The histological examination was completed by a pathologist. This examination was double-blinded. The crypt depth was measured in 20 microscopic fields. The crypts that extended perpendicularly from the mucosae to the muscular layer were measured. Twenty crypts were measured per slide. The percentage of mucosal thickness was calculated as the mucosal thickness divided by the total bowel wall thickness multiplied by 100. The percentage of goblet cells was calculated as the number of goblet cells divided by the number of all epithelial cells multiplied by 100. One hundred epithelial cells were measured in every slide. The number of Paneth cells in every slide was counted.

### Measurement of α-amylase and Na^+^-dependent bile salt transporter

The specimens were preserved at temperatures below 25° Celsius and humidity below 70 percent. Sections were cut at 5 μm thickness. The following procedures, including drying, baking, and immunohistochemical staining, were performed in accordance with a streptavidin horseradish peroxidase detection protocol. The antibodies against α-amylase and Na^+^-dependent bile salt transporters were provided by Servicebio (Wuhan, China). The slides were examined by OLYMPUS fluorescence microscopy. The stain intensity was measured by Image-Pro Plus 6.0 software. The staining with average intensity was assessed in 5 random fields using 400 × magnification.

### Statistics

All data are presented as the mean ± standard error. An independent-sample t-test was used for the analyses between the two groups. When significant differences between the mean values were detected, multiple comparison analyses were performed using the least significant difference test (SPSS Version 23, IBM Corp., Armonk, NY, USA). Weights, crypt depths, mucosal thickness, the percentage of goblet cells, and α-amylase and Na^+^-dependent bile salt transporter levels were compared using Student’s unpaired t-test (SPSS). A P value < 0.05 was considered a significant difference.

### Ethics statement

The rats used in the study were cared for in accordance with the institutional guidelines of Guangdong Medical Laboratory Animal Center. The study was granted permission by the Ethics Committee of Guangdong Medical Laboratory Animal Center.

## Results

**Figure 3:**
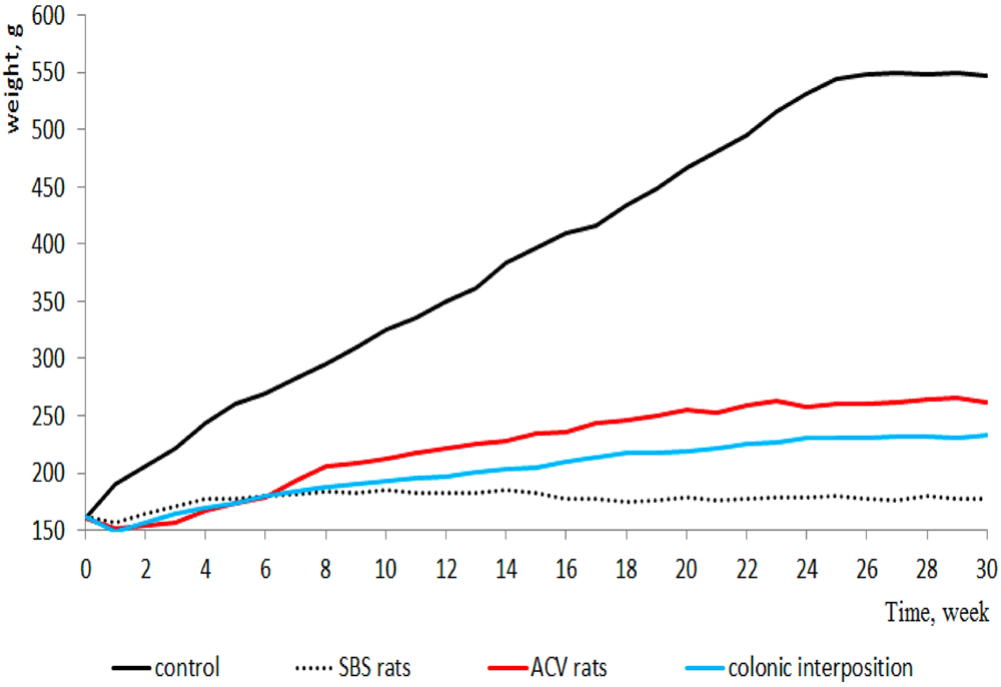
The weights of rats in all groups.

All rats in the control group and the SBS group survived. The survival rate of the ACV rats was 95 percent (one rat died of bowel obstruction, and another rat was added). The survival rate of the rats in the CI group was 80 percent (two rats died of bowel obstruction, two rats died of bowel perforation, and another four rats were added). Thirty weeks later, the weight of the rats in the SBS group was 177 ± 4 g and was much lighter than that of the rats in the control group (547 ± 19 g, P < 0.0001). Within 6 weeks, the weight of the ACV rats was lower than that of the rats in the SBS group. Thirty weeks later, the weight of the ACV rats (262 ± 13 g) was heavier than that of the rats in the SBS group (P < 0.0001). The weight of the rats in the CI group (233 ± 12 g) was heavier than that of the rats in the SBS group but was lighter than that of the ACV rats (P < 0.0001). The weights of the rats in all groups are illustrated in Fig. 3.

**Figure 4:**
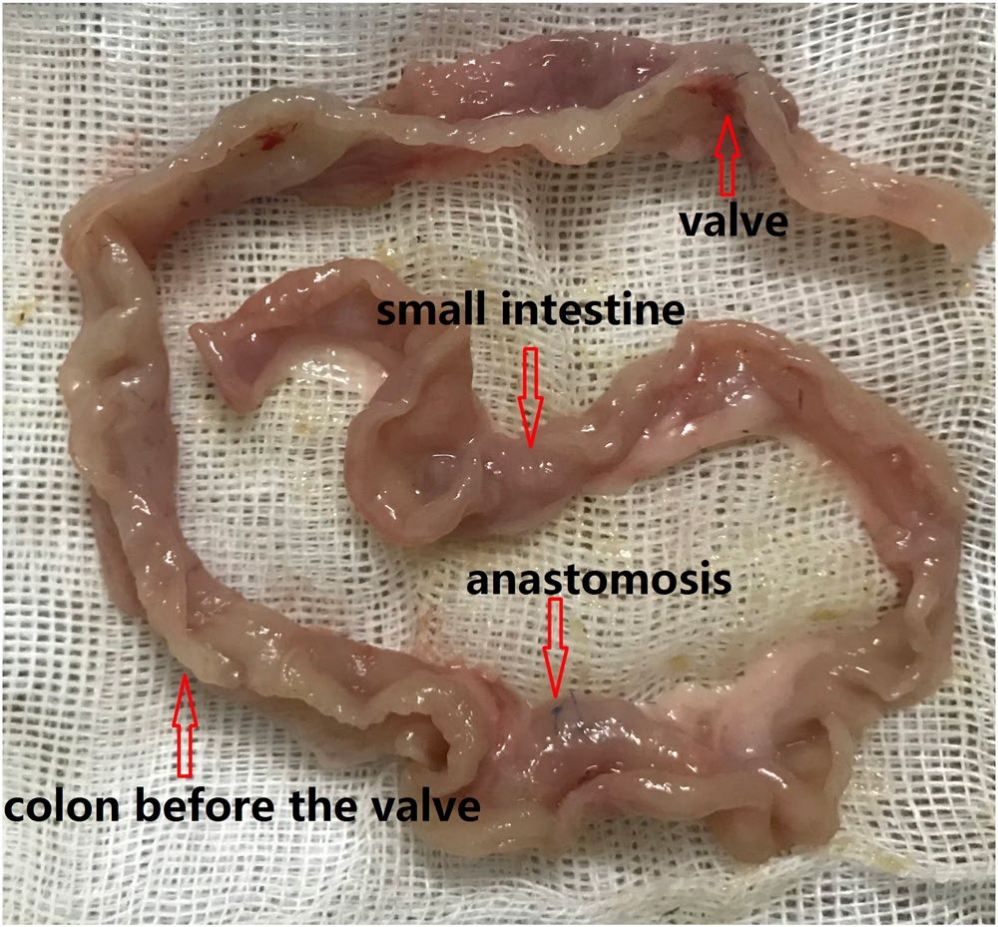
Gross examination of ACV rats. Colon before the valve was larger and thicker.

**Figure 5:**
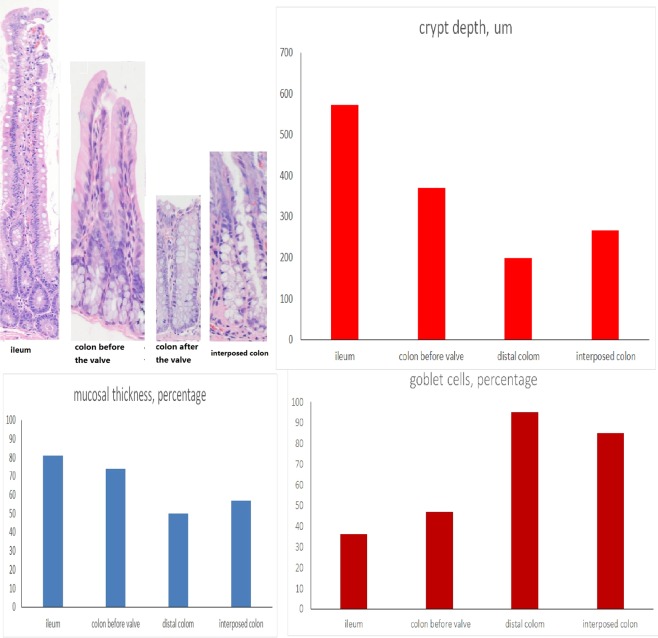
Microscopic examination of ileum, colon before valve in ACV rat, colon after valve in ACV rat and interposed colon in CI rat. The colon before valve had deeper crypt depth, thicker wall and fewer goblet cells than that after valve.

**Figure 6:**
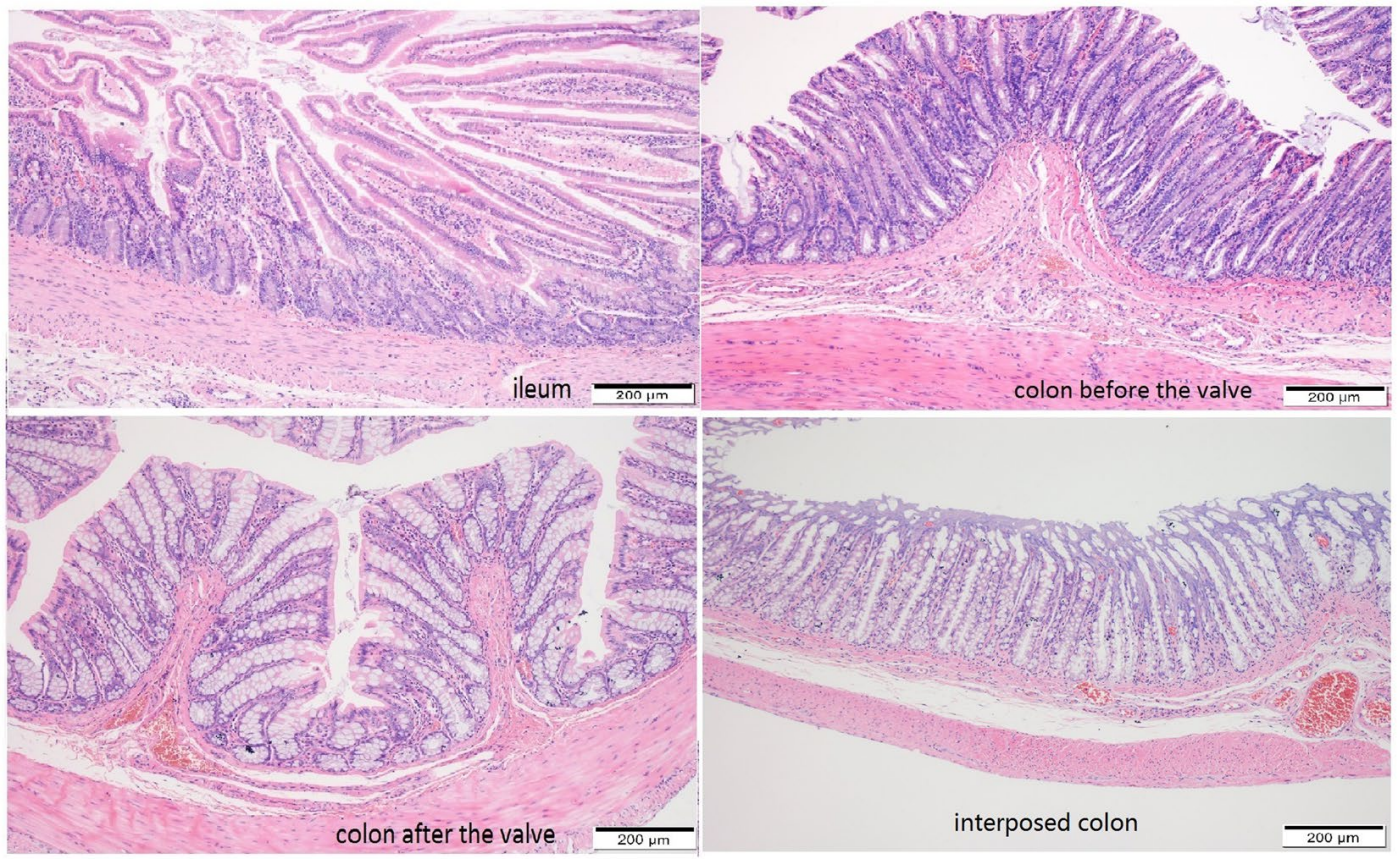
The metaplasia changes were seen in the colon before the valve. They had less goblet cells, more active crypt nuclei, and “saw-toothed” crypts.

**Figure 7:**
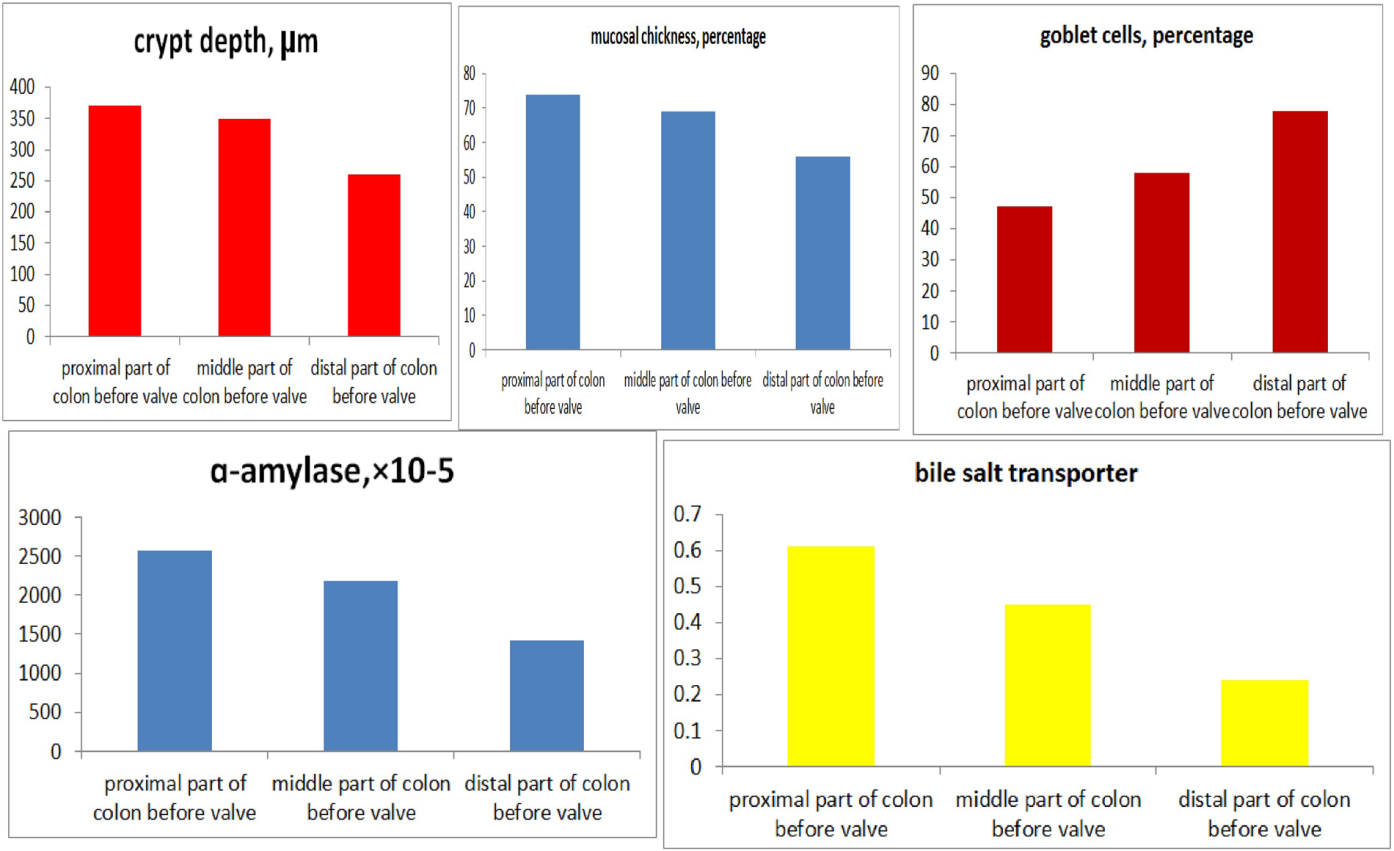
Microscopic, α-amylase and Na + -dependent bile salt transporter in different parts of colon before the valve.

**Figure 8:**
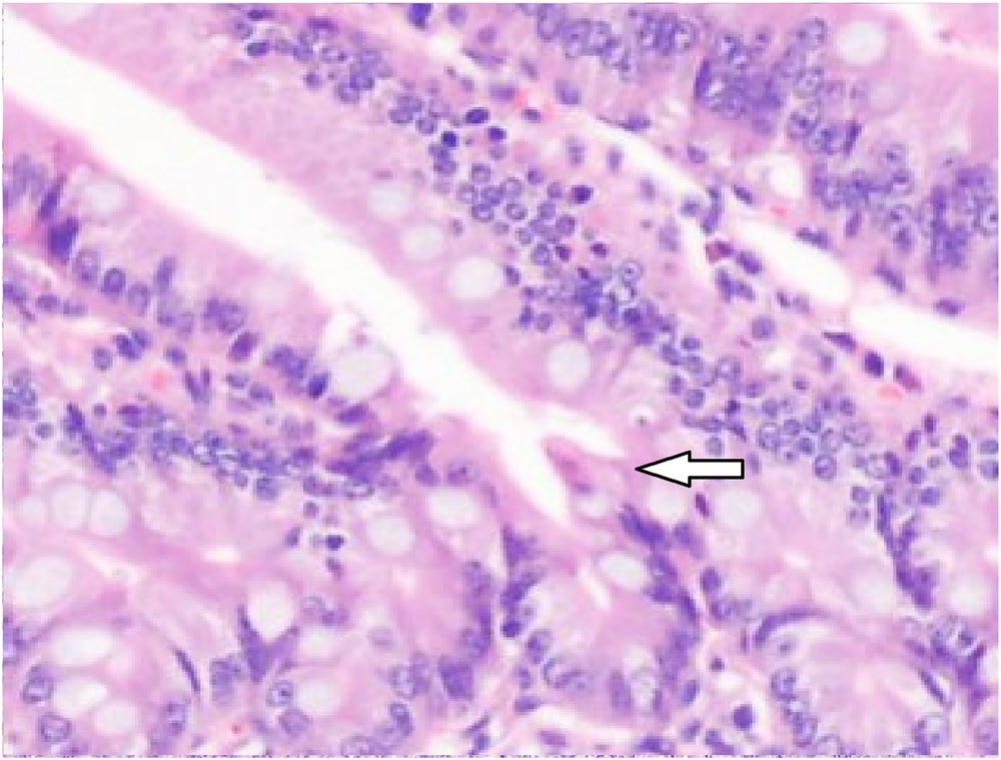
Paneth cell was seen in the colon before the valve.

The gross histological examination found that the colons before the valves had a larger colonic lumen and thicker bowel wall than the colons after the valves (Fig. 4). Upon microscopic examination, the colons before the valves had deeper crypts, thicker mucosa and fewer goblet cells than the colons after the valves (P < 0.001, Fig. 5). Though the colons before the valves did not completely resemble the small intestine, their epithelial cells had more active crypt nuclei and “saw-toothed” crypts, called epithelial metaplasia (Fig. 6). In regards to the metaplasia of the colons before the valves, areas closer to the small intestine showed more obvious metaplasia than areas farther from the small intestine (Fig. 7). The interposed colons of the rats in Group 4 also had some form of colonic adaptation, but not as much as the colons before the valves in the ACV group (P < 0.001, Figs. 5, 6 and 7). Paneth cells were found in the colons before the valves in the ACV rats (Fig. 8). However, no Paneth cells were found in the interposed colons of the rats in the CI group.

**Figure 9:**
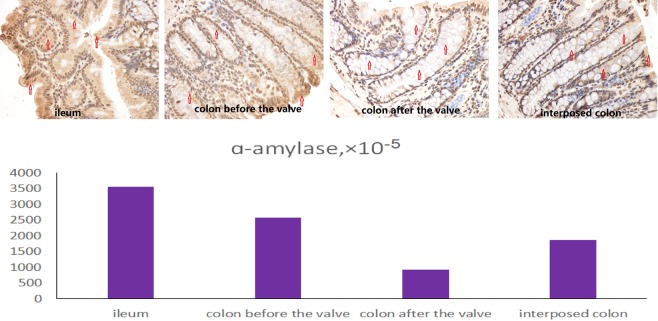
Immunohistochemistry staining of α-amylase in ileum, colon before valve in ACV rat, colon after valve in ACV rat and interposed colon in CI rat.

**Figure 10:**
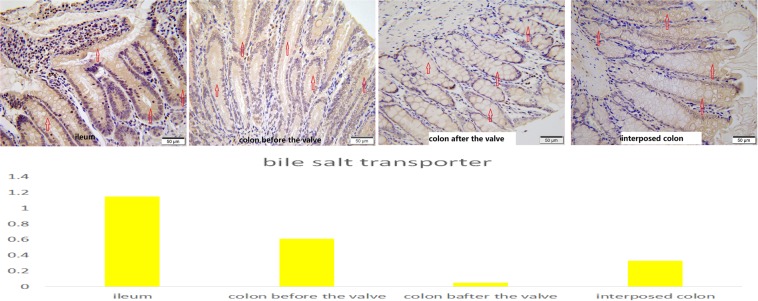
Immunohistochemistry staining of Na + -dependent bile salt transporter in ileum, colon before valve in ACV rat, colon after valve in ACV rat and interposed colon in CI rat.

For the ACV rats, the level of α-amylase in the colons before the valves was higher than that of the colons after the valves, higher than that of the colons in the control group and the SBS group, and higher than that of the interposed colons in the CI group, but lower than that of the ileum (Fig. 9). In the colons before the valves, the areas closer to the small intestine showed more α-amylase expression than those farther from small intestine (P < 0.05, Fig. 7). The results for the Na^+^-dependent bile salt transporter were similar to those of α-amylase (Fig. 10).

## Discussion

Short bowel syndrome (SBS) is a clinical syndrome caused by an overly short small bowel that cannot adequately meet the requirements of the body for fluid and nutrients. Some patients who need long-term intravenous nutrients require surgery. The surgical procedure used in these patients depends on their actual conditions. Common histological changes in SBS are segmental bowel dilatation, poor peristalsis and rapid transit. Bowel dilatation occurs in tapering enteroplasty with a mechanical stapling device^8^. For patients with a critically short bowel, the longitudinal intestinal lengthening and tailoring (LILT) procedure^1^ and serial transverse enteroplasty (STEP) are the two most common procedures.^2^ These two procedures can achieve similar results.^7^ The segmental reversal of the small bowel^3^ and iso-peristaltic colonic interposition^4^ are the two procedures used to slow the intestinal transit time. The sequential lengthening procedure and controlled tissue expansion (CTE) procedure are the two procedures that can increase the absorptive area.^5^ Distraction enterogenesis has been proven in animal experiments to increase the absorptive area.^6^ Intestinal transplantation is a challenging operation with a fair outcome in SBS patients.^7^ The enhancement of the absorptive function of the residual bowel is another treatment method for SBS patients^9,10^. Most recent studies have focused on exogenous growth factors, including somatostatin,^11^ glutamine and growth hormone,^12^ epidermal growth factors,^13^ insulin-like growth factor-I,^14^ glucagon-like peptide 2,^15^ and hepatocyte growth factors.^16^ However, for some SBS patients, the residual intestines are critically short, therefore the outcomes are not very satisfactory. It is known that the ileocaecal valve may enable the small bowel to have more contact with the bowel content by slowing the transit time.^17^ Though the ileocaecal valve is important,^18,19^ the artificial small bowel will not result in functional improvements.^20^

In addition to the small bowel, we can focus on another part of the bowel, the colon. Enabling the colon to have absorptive function similar to that of small bowel is not a new concept. A previous study revealed that the colon in a small bowel environment shows some small bowel features, such as a deepening of crypt depth, a thickening of the mucosal wall and increasing maltase levels.^21^ Kono K^22^ described a case in which an interposed colon had some form of small bowel features 24 months after colonic interposition. Colonic interposition is not an ideal procedure for SBS patients because of its complicated manipulation and the likelihood of complications. To slow the transit time and enhance the colonic absorptive function, we shifted the position of the ileocaecal valve from the caecum to the colon. Similar to most studies,^23^ we used SD rats for this study. We created a valve in the colon. Thus, most of the time, the bowel content could not pass the valve, and the colon had more opportunity to contact the small bowel environment (the ileocaecal valve was removed). The transit time in the small bowel became longer as if it was caused by the ileocaecal valve. The colonic valve was elastic. As the bowel content accumulated, the valve would expand so that the stool could pass through it.

Some positive results were achieved. The ACV rats were heavier than those in the SBS group and the CI group. The histological results showed that the change in the colon before the valve was obvious, including a thicker mucosa, deeper crypts and fewer goblet cells. Paneth cells represent a unique marker of the small bowel, and their function is involved in the regulation of bowel bacteria.^24^ Paneth cells were found in the colons before the valves in this experiment. Furthermore, α-amylase is an indicator of the absorptive function of starch, and the Na^+^-dependent bile salt transporter involves the absorption of bile by the ileum.^25^ The results of this study revealed that the α-amylase and Na^+^-dependent bile salt transporter levels in the colons before the valves were higher than those in the colons after the valves, showing that the colons before the valves had an absorptive function similar to that of the small bowel. The study also found that the area proximal to the valve showed more change, though all parts of colon do have that potential.^21^ In this study, after a valve was created in the colon, the proximal colon had more opportunity to contact the small bowel environment, resulting in more changes. It is likely that ACV creation in the distal colon is better than creation in the middle colon because it allows a larger number of changes. However, more tests and clinical trials are needed to verify which part of the colon is better for the creation of the valve.

Colon interposition (CI) can also improve the colonic absorptive function of interposed colons.^21^ This study found that the colonic absorptive function of the ACV rats was better than that of the CI rats, and no Paneth cells were seen among the interposed colon of CI rats. After CI, the bowel content passed through the interposed colon at the same speed as before CI. Therefore, the interposed colon did not have more chance to contact the small bowel environment than the colon before the valve in the ACV rats, leading to less change. Compared with the three anastomoses during the CI, the ACV needs no anastomosis. According to the results of this study, the possibility of complications with ACV surgery, such as obstruction and perforation, is lower than that of CI.

In this experiment, the weights of the ACV rats were lighter than those in the SBS group within the first six weeks after surgery. The rats needed some time for the colonic epithelium to acquire some absorptive function similar to that of the small bowel. If the ACV procedure is allowed to be performed on humans, concern about the time needed for colonic change to occur should be minimal because of the application of IV fluid and nutritional supplementation. Furthermore, because the rats’ large caecum has a powerful function of absorbing water, the caecum was cut in the experiment to avoid obstruction caused by hard stool. Thus, the stool could become looser and pass through the valve more easily. For humans, the caecum is not that large, and the content in the proximal colon is semisolid, therefore it will pass through the valve more easily. Thus far, it is unclear whether obstruction caused by the caecum will be a real problem for humans. Furthermore, the valve created in this study is probably not as perfectly functional as a real ileocaecal valve. Therefore, the improvement of the technique for creating a better valve will be a future research focus.

The colon should not be viewed simply as a channel that transports stool. In this experiment, the colon before the valve had histological results and absorptive functions that were similar to those of the small bowel, and it was a very enhanced colonic absorptive function. Compared with CI, the ACV procedure is quite simple (it requires no anastomosis), and it has fewer complications. The ACV procedure is better than CI for the short-gut rat. More time is needed to determine whether the colon can be transformed and function similar to a real small bowel. If a similar result can be achieved in humans, SBS patients may gain weight, have improved absorptive function and depend less on parenteral nutrition.

## Data Availability

The authors declare that supporting data will be available to the Editorial Board Members and referees at the time of submission for the purposes of evaluating the manuscript. The authors can provide adequate assurances that can comply with the publication’s requirements for sharing materials.
